# Supplementary material for: The incidence and predictors of peripheral arterial disease among type 2 diabetes mellitus patients at Felege Hiwot Comprehensive Specialized Hospital, Northwest Ethiopia, 2023: A retrospective follow-up study
Source: PLoS One. 2025 Apr 1;20(4):e0320948. doi: 10.1371/journal.pone.0320948 (PMC11961006; doi:10.1371/journal.pone.0320948)
Supplement: Attachment — Submitted filename: Response_to_Reviewers_auresp_2.docx [file pone.0320948.s003.docx]

January 31, 2025

**To: PLOS ONE Editorial Office**

**Authors’ point-by-point Responses to a Manuscript ID: PONE-D-23-41579R1**

**Dear Editor in Chief, Greetings.**

We have submitted these authors' responses to peer-review comments and questions for a manuscript entitled: The incidence and predictors of peripheral arterial disease among type 2 diabetes mellitus patients at Felege Hiwot Comprehensive Specialized Hospital, Northwest Ethiopia, 2023: a retrospective follow-up study.

Dear Editor, first, we would like to thank the PLOS ONE Editorial Office members, especially the chief editor, who timely assigned the competent academic editor and facilitated the progress of the review forum. Second, our special thanks go to the academic editor for his editorial contribution and for assigning the potential skilled and experienced reviewers in the field promptly. Moreover, our deepest gratitude goes to the esteemed reviewers for their constructive comments and scientific contributions that helped us improve the quality of this manuscript.

Dear academic Editor and reviewers, we have made the necessary corrections and responses to those comments raised by reviewers. Accordingly, we have added the authors’ responses in the review forum, as well as the revised manuscript and the tracked changes to the manuscript on the web page.

With regards!

Gebrie Getu Alemu

[gebryegetu27@gmail.com](mailto:gebryegetu27@gmail.com)

University of Gondar, Ethiopia

PO. Box 196

Corresponding Author

**Table:** **Authors’ responses to editor and peer-review of reviewer**

| **Academic Editor's comments and Authors’ responses** | | |
| --- | --- | --- |
| **Academic Editor's comments** | | **Authors’ Responses** |
| **Journal Requirements** | |  |
| Journal Requirements:  Please review your reference list to ensure that it is complete and correct. If you have cited papers that have been retracted, please include the rationale for doing so in the manuscript text, or remove these references and replace them with relevant current references. Any changes to the reference list should be mentioned in the rebuttal letter that accompanies your revised manuscript. If you need to cite a retracted article, indicate the article’s retracted status in the References list and also include a citation and full reference for the retraction notice. | | Dear editor, thank you for the comment. We have reviewed all references cited in the manuscript. All cited references are correct and complete. We haven’t cited any retracted papers. |
| **Reviewer 3 comments and Authors’ responses** | | |
| **Reviewer 3 comments** | **Authors’ Responses** | |
| In the abstract – Result sub-section: “The incidence rate of peripheral arterial disease was high. “The authors need to specify the reference to conclude high like compare with the recommended value or compare with the previous study or ---- | Dear reviewer thank you for your comment. We have said the incidence rate of peripheral arterial disease in our study was high by comparing with the previous studies conducted elsewhere.  The incidence rates of peripheral arterial disease in the following studies (with the given link below) were less than ours. So we said that our study incidence rate of peripheral arterial disease was high by comparing with these studies.  <https://journals.sagepub.com/doi/full/10.1177/2150132720957449>  <https://www.sciencedirect.com/science/article/abs/pii/S1056872714001287>  We have already discussed and cited this in the discussion section. Kindly see **lines from 326-340.** | |
| In the result section  The hazard of PAD in female type 2 DM patients was twofold (AHR = 2.18), more than in males. Furthermore, the hazard of PAD among type 2 DM patients with baseline FBS > 140 mg/dl was 3.3 times higher than those with FBS ≤ 140 mg/dl (AHR = 3.34). The authors need to include 95%CI. | Dear reviewer, we appreciate your comment and we have corrected the comments. The changes can be seen **page 16, lines 280-283** | |
