# Supplementary material for: The incidence and predictors of peripheral arterial disease among type 2 diabetes mellitus patients at Felege Hiwot Comprehensive Specialized Hospital, Northwest Ethiopia, 2023: A retrospective follow-up study
Source: PLoS One. 2025 Apr 1;20(4):e0320948. doi: 10.1371/journal.pone.0320948 (PMC11961006; doi:10.1371/journal.pone.0320948)
Supplement: Attachment — Submitted filename: Response to Reviewers.docx [file pone.0320948.s002.docx]

**August 05, 2024**

**To: PLOS ONE Editorial Office**

**Authors’ point-by-point Responses to a Manuscript ID: PONE-D-23-41579**

**Dear Editor in Chief, Greetings.**

We have submitted these authors' responses to peer-review comments and questions for a manuscript entitled: The incidence and predictors of peripheral arterial disease among type 2 diabetes mellitus patients at Felege Hiwot Comprehensive Specialized Hospital, Northwest Ethiopia, 2023: a retrospective follow-up study.

Dear Editor, first, we would like to thank the PLOS ONE Editorial Office members, especially the chief editor, who timely assigned the competent academic editor and facilitated the progress of the review forum. Second, our special thanks go to the academic editor for his editorial contribution and for assigning the potential skilled and experienced reviewers in the field promptly. Moreover, our deepest gratitude goes to the esteemed reviewers for their constructive comments and scientific contributions that helped us improve the quality of this manuscript.

Dear academic Editor and reviewers, we have made the necessary corrections and responses to those comments raised by reviewers 1 and 2 point by point, page by page, and line by line sequentially. Accordingly, we have added the authors’ responses in the review forum, as well as the revised manuscript and the tracked changes to the manuscript on the web page.

With regards!

Gebrie Getu Alemu

[gebryegetu27@gmail.com](mailto:gebryegetu27@gmail.com)

University of Gondar, Ethiopia

PO. Box 196

Corresponding Author

**Table:** **Authors’ responses to peer-review of reviewers 1 and 2 comments**

| **Academic Editor's comments and Authors’ responses** | | | |
| --- | --- | --- | --- |
| **Academic Editor's comments** | | **Authors’ Responses** | |
| **Journal Requirements** | |  | |
| 1. Please ensure that your manuscript meets PLOS ONE's style requirements, including those for file naming. The PLOS ONE style templates can be found at   <https://journals.plos.org/plosone/s/file?id=wjVg/PLOSOne_formatting_sample_main_body.pdf> and  <https://journals.plos.org/plosone/s/file?id=ba62/PLOSOne_formatting_sample_title_authors_affiliations.pdf> | | Dear Editor, thank you very much for your suggestion. We have confirmed that the manuscript format is based on the PLOS ONE style. | |
| 2. We note that your Data Availability Statement is currently as follows: All relevant data are within the manuscript and its Supporting Information files.   Please confirm at this time whether or not your submission contains all raw data required to replicate the results of your study. Authors must share the “minimal data set” for their submission. PLOS defines the minimal data set to consist of the data required to replicate all study findings reported in the article, as well as related metadata and methods (<https://journals.plos.org/plosone/s/data-availability#loc-minimal-data-set-definition>).  For example, authors should submit the following data:  - The values behind the means, standard deviations and other measures reported; - The values used to build graphs;  - The points extracted from images for analysis. Authors do not need to submit their entire data set if only a portion of the data was used in the reported study. If your submission does not contain these data, please either upload them as Supporting Information files or deposit them to a stable, public repository and provide us with the relevant URLs, DOIs, or accession numbers. For a list of recommended repositories, please see <https://journals.plos.org/plosone/s/recommended-repositories>.If there are ethical or legal restrictions on sharing a de-identified data set, please explain them in detail (e.g., data contain potentially sensitive information, data are owned by a third-party organization, etc.) and who has imposed them (e.g., an ethics committee). Please also provide contact information for a data access committee, ethics committee, or other institutional body to which data requests may be sent. If data are owned by a third party, please indicate how others may request data access. | | Dear Editor, thank you for this comment. All relevant data are within the manuscript. | |
| 3. When completing the data availability statement of the submission form, you indicated that you will make your data available on acceptance. We strongly recommend all authors decide on a data sharing plan before acceptance, as the process can be lengthy and hold up publication timelines. Please note that, though access restrictions are acceptable now, your entire data will need to be made freely accessible if your manuscript is accepted for publication. This policy applies to all data except where public deposition would breach compliance with the protocol approved by your research ethics board. If you are unable to adhere to our open data policy, please kindly revise your statement to explain your reasoning and we will seek the editor's input on an exemption. Please be assured that, once you have provided your new statement, the assessment of your exemption will not hold up the peer review process. | | Dear Editor, Thank you for your recommendations.  All authors have decided about data sharing. We assured that our entire data will be freely accessible if our manuscript will be accepted for publication. | |
| **Reviewer 1 comments and Authors’ responses** | | | |
| **Reviewer 1 comments** | **Authors’ Responses** | | |
| 1. There are linguistic and editing errors like lack of hyphens, periods, inconsistent capitalization, misspellings, and formatting inconsistencies. | Dear reviewer, thank you for providing your feedback and comments on our manuscript. Your detailed feedback and comments on our manuscript is too much appreciated. We have revised the manuscript entirely. Linguistic and editing errors have been corrected. We have carefully improved the grammar of the manuscript by applying a Grammarly tool. | | |
| 2. Can you provide more specific details on the existing research gap in Ethiopia regarding PAD and vascular complications in type 2 diabetes mellitus patients? How does the proposed study aim to address this gap, and what unique contributions does it offer to the existing literature? | Dear reviewer, thank you for your suggestions and questions. We have tried to incorporate the research gap on the existing knowledge in Ethiopia regarding to PAD and vascular complications. **Pages 4-5, line 86-92.**  The aim of the study how to address the gap and contribution is presented at the last paragraph of the background **page 5, line 92-98.** | | |
| 3. You focused your study on type 2 diabetic patients aged 14 years and older. Could you explain why you didn't include children under 14, considering that type 2 diabetes is also affecting younger individuals? Additionally, what's the rationale behind choosing this particular age as the cutoff point? | Dear reviewer, the reason behind why we included adults than children is that because type 2 DM affects adults more than children in our setups. Children are very rarely affected by type 2 DM but they are affected by type 1 DM. The reason behind choosing age 14 as cutoff point is that children below age of 14 are considered to be part of pediatric population, not adults in clinical practice and their follow up is different from the adults. So for generalizability we had chosen the most affected and vulnerable homogenous adult population. | | |
| 4. It would be better if the discussion section discusses potential differences in study populations, methodologies, and healthcare systems that may contribute to variations in findings. Additionally, considering factors such as sample size and follow-up duration could provide further context for interpretation. | Dear reviewer, thank you for your suggestions and comments. The comments are constrictive and invaluable. We have tried to discuss potential differences in study populations, methodologies, and healthcare systems, sample size and follow-up duration. We have discussed and revised according to the comments and incorporated in the discussion section accordingly in the revised manuscript. | | |
| 5. The discussion addresses variations in PAD incidence rates across different studies and attributes the higher incidence rate in this study to differences in case detection methodologies. However, it would be helpful to discuss potential limitations of Doppler ultrasound evaluations in detecting PAD, such as operator dependence and variability in interpretation, and whether alternative diagnostic approaches could provide complementary information. | Dear reviewer, thank you for your comments. The potential limitations of Doppler ultrasound evaluations in detecting PAD have been incorporated in the limitation part of the discussion. This can be seen **page 19, line 357-362.** | | |
| 6. Regarding the predictors identified for PAD development in type 2 DM patients, how could healthcare professionals integrate this information into clinical practice to improve patient care and outcomes? | Dear reviewer, thank you for your question. We have disseminated this study to the responsible bodies and stake holders. We have been submitted the hard and soft copies to Bahir Dar university college of medicine and health sciences, Felege Hiwot comprehensive specialized referral hospital and Amhara public health institute. We have also presented at different seminars and workshops. So the health professionals can easily accessed the study and we have recommended the findings of this study to the health care professionals to act accordingly. **Page 19-20, line 367-374** | | |
| 7. Are there any unique challenges or opportunities in Ethiopia's healthcare system or socio-economic environment that should be considered when interpreting the study findings and implementing interventions? | Dear reviewer, thank you for the question. Ethiopia’s healthcare system and socio-economic level are very challenging as follows.  System challenging   1. **Access to care:** many patients, especially in rural areas lack access to healthcare, diabetic education, and necessary medications. 2. **Healthcare Infrastructure**:   Inadequate healthcare infrastructure can impede effective diabetes management. This includes insufficient medical facilities, lack of diagnostic tools, and poor distribution of healthcare resources.   3. **Coordination of Care**: Managing diabetes often requires coordinated care among various healthcare providers, including primary care physicians, endocrinologists, dietitians, and other specialists. Lack of coordination can lead to fragmented care.   **Socioeconomic Challenges**   1. **Cost of Treatment**: Diabetes management can be expensive due to the cost of medications, regular monitoring supplies, and healthcare visits. This financial burden can be prohibitive for many patients. 2. **Health Disparities**: Socioeconomic factors such as income, education level, and access to healthy food can significantly impact diabetes management. Disparities can lead to unequal outcomes among different populations. 3. **Insurance Coverage**: Limited or no health insurance can restrict access to necessary diabetes care and medications, leading to poor management and control of the disease.   These are the challenge which potentially affects to implement the interventional strategies in Ethiopia. | | |
| **Reviewer 2 comments and Authors’ responses** | | | |
| **Reviewer 2 comments** | | | **Authors’ Responses** |
| In the abstract line 40 Result: please write as Results  Being female (AHR=2.18(1.36, 3.51), age above 65 years (AHR=1.66, CI: 1.06, 2.61), and fasting blood sugar of more than 140mg/dl 43 (AHR= 3.34(1.62, 6.90) were predictors for time to peripheral arterial disease in type 2 diabetes mellitus.( Line 42- 44)  Comments: AHR: Please write the entire word (definition) first, followed by its abbreviation in brackets, and then utilize it throughout your write up. Please make the same corrections for the remaining abbreviations in this document. Please add 95% CI consistently | | | Dear reviewer thank you for your comments. We have corrected these accordingly. |
| In the introduction section (line 77)  Some studies have estimated the median times for onset of PAD in type2 diabetes mellitus patients Comments: type2 correct it as type 2  There are factors which can affect the survival of type 2 DM patients from PAD occurrence. (Line 79) Comments: DM: Please write the entire word (definition) first, followed by its abbreviation in brackets, and then utilize it throughout your write up. Please make the same corrections for the remaining abbreviations in this document. | | | Dear reviewer, We also appreciate your comment and we have corrected it. |
| The material and methods is Ok, just to delete some redundancy sentences Comments: Please delete the followings in the material and methods section. Study design (line 99)  An institution-based retrospective follow-up study was conducted at Felege Hiwot Comprehensive Specialized Hospital, Northwest Ethiopia (line 100-101) Please rewrite as Study design, area and period in line 102 Please add Independent variables as a subtitle after line 153 | | | Dear reviewer, Thank you for picking the important strengths and supporting insight of the methods and materials. We have  removed and modified the redundancy sentences  accordingly. Study design, area and period have been rewritten as per recommendation. **Page 5, line 100.** |
| Results The cumulative survival to PAD probabilities of T2DM patients after 5, 10 and 15 years were 0.97, 0.76, 0.35 respectively.(Lines 261-262) Comments: please rewrite the above sentence as The cumulative survival probabilities of PAD among T2DM patients after 5, 10 and 15 years were 0.97, 0.76 and 0.35, respectively. Please rewrite the sentence in line 280 as (AHR=1.66, 95% CI: 1.06, 2.61) higher as compared to those whose age was ≤65 years. | | | Dear reviewer, your comments are constructive. We have been rewrite as per the recommendations. The changes can be seen **page 13, line 247-248page line and 15, line 278.** |
| Discussion The importance or implication or significance of each result should be stated in the discussion section. | | | Dear reviewer, thank you for your invaluable comments. We have incorporated the implications of the study in the discussion section under the sub-headings of public health and research implications. It can be seen **Page18-19, line 339-349** |
| In the Conclusion section do not use figures (numbers) rather conclude the main findings. | | | Dear reviewer, we acknowledge your  constructive comments and feedbacks. We have corrected it accordingly. |
| Figures 1- 4 needs edition for example analysis time by what? month or year? Clearly state it. Chi2 please replace it by X2 | | | Dear reviewer, we appreciate your comments. We have modified as per the recommendations. The changes can be seen in the **figures (1-4).** |
