# Supplementary material for: The incidence and predictors of peripheral arterial disease among type 2 diabetes mellitus patients at Felege Hiwot Comprehensive Specialized Hospital, Northwest Ethiopia, 2023: A retrospective follow-up study
Source: PLoS One. 2025 Apr 1;20(4):e0320948. doi: 10.1371/journal.pone.0320948 (PMC11961006; doi:10.1371/journal.pone.0320948)
Supplement: Attachment — Submitted filename: Response_to_Reviewers_auresp_4.docx [file pone.0320948.s005.docx]

February 22, 2025

**To: PLOS ONE Editorial Office**

**Authors’ point-by-point Responses to a Manuscript ID: PONE-D-23-41579R3**

**Dear Editor in Chief, Greetings.**

We have submitted these authors' responses to editor’s comments for a manuscript entitled: The incidence and predictors of peripheral arterial disease among type 2 diabetes mellitus patients at Felege Hiwot Comprehensive Specialized Hospital, Northwest Ethiopia, 2023: a retrospective follow-up study.

Dear Editor, first, we would like to thank the PLOS ONE Editorial Office members, especially the chief editor, who timely assigned the competent academic editor and facilitated the progress of the review forum. Second, our special thanks go to the academic editor for his editorial contribution and for assigning the potential skilled and experienced reviewers in the field promptly.

Dear academic Editor we have made the necessary corrections and responses to those comments raised by editors. Accordingly, we have added the authors’ responses in the review forum, as well as the revised manuscript and the tracked changes to the manuscript on the web page.

With regards!

Gebrie Getu Alemu

[gebryegetu27@gmail.com](mailto:gebryegetu27@gmail.com)

University of Gondar, Ethiopia

PO. Box 196

Corresponding Author

**Table:** **Authors’ responses to editors**

| **Academic Editor's comments and Authors’ responses** | |
| --- | --- |
| **Editor's comments** | **Authors’ Responses** |
| **Journal Requirements** |  |
| Journal Requirements:  Please review your reference list to ensure that it is complete and correct. If you have cited papers that have been retracted, please include the rationale for doing so in the manuscript text, or remove these references and replace them with relevant current references. Any changes to the reference list should be mentioned in the rebuttal letter that accompanies your revised manuscript. If you need to cite a retracted article, indicate the article’s retracted status in the References list and also include a citation and full reference for the retraction notice. | Dear editor, thank you for the comment. We have reviewed all references cited in the manuscript. All cited references are correct and complete. We haven’t cited any retracted papers. |
| We understand that the Grammarly tool was used to assist you with this revision, but unfortunately language errors remain throughout the manuscript. The manuscript still requires copyediting in order to improve the English grammar and syntax to a publishable standard. We recommend asking a native English-speaking colleague to assist you or to enlist the help of a professional copyediting service. | Dear editor, thank you for the comment. We have carefully checked and corrected the grammatical errors, punctuations and spelling mistakes of the overall manuscript. We have gotten a native English-speaking colleague assistant and we have applied the professional copyediting services to improve the overall language and quality of writing throughout our manuscript. We have made our best to improve the overall language quality and writing of the manuscript. At this stage, we believe that the language of the manuscript meets the publishable standards. Kindly see the updated and the track changed manuscripts. |
